# Identification of Selective Sweeps in the Domesticated Table and Wine Grape (*Vitis vinifera* L.)

**DOI:** 10.3389/fpls.2020.00572

**Published:** 2020-05-14

**Authors:** Ling Kui, Min Tang, Shengchang Duan, Shouling Wang, Xiao Dong

**Affiliations:** ^1^State Key Laboratory for Conservation and Utilization of Bio-Resources in Yunnan, Yunnan Agricultural University, Kunming, China; ^2^Dana-Farber Cancer Institute, Harvard Medical School, Boston, MA, United States; ^3^Institute of Life Sciences, Jiangsu University, Zhenjiang, China; ^4^Nowbio Biotechnology Company, Kunming, China

**Keywords:** *Vitis vinifera* L., next-generation sequencing, QTLs, domestication signal, SNPs, selective sweep

## Abstract

Grapevine (*Vitis vinifera*) is one of the most important fruit species in the Classical Mediterranean world. It is thought to have been domesticated 6,000–8,000 years ago in the Near East. However, the domestication of its wild relative into wine grapes or table grapes remains largely unknown. In this study, we analyzed 30 table grapes, 30 wine grapes, 30 dual-purpose grape accessions, as well as 30 wild relatives (*Vitis vinifera* ssp. *sylvestris*). The phenotypic comparison showed striking differences in berry weight, acidity and the content of aroma. Based on a total of 7,522,958 single-nucleotide polymorphisms, we identified several significant selective sweep regions for table and wine grapes. Besides the well-known sex-determination locus on chromosome 2, the other four highest signals shared by table and wine grapes could not be linked to the known QTLs. The identification of these genomic regions under selection sweep may reveal agronomically important traits that have been selected during grape domestication. This information not only sheds light on the mechanisms of adaptions and diversification, but also guide the genetic improvement in breeding programs.

## Introduction

Approximately 13,000 years ago, human began to domesticate and selectively breed crops (Smith, 2001; Diamond, 2002). During this process, wild species were genetically selected on certain traits that are advantageous for human as sources of food or materials. The most remarkable evolutionary transitions are enlarged seed and fruit sizes (Frary et al., 2000), seed dispersal, loss of dormancy, and flowering and ripening time (Cockram et al., 2007). Meantime, the notably diversification in plant architecture (Clark et al., 2004), adaptation and quality can also be observed. In addition to phenotypes, plant domestication also leaves genetic signatures on both population structure and genetic diversity of the domesticated species (Doebley et al., 2006; Abbo et al., 2014). Understanding how plants are affected during the domestication process, not only can shed light on the mechanisms of adaptions and diversification (Diamond, 2002) but also will guide the genetic improvement in breeding programs.

As one of the oldest domesticated crop plants, grapevine (*Vitis vinifera* L.) is now economically the most important cultivated fruit crop in the world (Alston and Sambucci, 2019). Since domesticated from its wild progenitor, *Vitis vinifera* ssp. *sylvestris*, grape was already a major fruit crop in the Mediterranean area. Moreover, the archeological record suggests that grape has been a source of food and wine 6000–8000 years ago in the Near East (McGovern et al., 2017; McGovern, 2019). Till now, several significant morphological shifts as a result of domestication have been reported, such as larger berry and bunch sizes (Cabezas et al., 2006), higher sugar content, increased variation in berry color (This et al., 2007) and a shift from dioecy to a hermaphroditic mating system (Carmona et al., 2007). Genes contributing to the morphological shifts during domestication and improvement of grapevines have been identified in several resequencing and genetic studies (Costantini et al., 2008; Fechter et al., 2012; Picq et al., 2014; Chen et al., 2015; Correa et al., 2016; Liang et al., 2019).

Generally, commercially cultivated grapes can be classified as either table or wine grapes based on their intended method of consumption: table grapes or wine grapes. Though almost all of them belong to the same species (*V. vinifera*), significant differences between table and wine grapes were brought about through selective breeding. Table grapes tend to have large, seedless fruit with relatively thin skin, while wine grapes are smaller, usually seeded and have relatively thick skins. At the time of harvest wine grapes tend to be very sweet (approximately 24% sugar by weight), while table grapes have usually around 15% sugar by weight. In addition, wine grapes are often small with concentrated flavors, whereas table grapes are large, bursting with a lot more water.

Genetic diversity among *V. vinifera* varieties has been studied. Simple sequence repeats were used by Bowers et al. (1999) and more recently, a chip containing 9,000 SNPs was applied on 950 *vinifera* and 59 *sylvestris* accessions by Myles et al. (2011). Their results suggested that grape domestication led to a mild reduction of genetic diversity, indicating that grape is a reasonable perennial model for studying the accumulation of deleterious variation in the absence of a pronounced bottleneck. In addition, whole-genome sequencing (WGS) was used in more recent studies to assess structural variation among grape varieties (Cardone et al., 2016; Xu et al., 2016). Di Genova et al. (2014) identified a large number of structural variants and SNPs by comparing genome of “Sultanina” with the reference genome of a nearly homozygous genotype of cultivar “Pinot noir” (PN40024), which are table and wine cultivar, respectively. Moreover, Migicovsky et al. (2017) combined phenotypic data and genome-wide polymorphism data of 580 table and wine grape accessions, and identified some large effect loci controlling phenotypic traits that have been targeted during domestication and breeding, such as hermaphroditism, lighter skin color and muscat aroma.

However, there has been little research performed to identify the positive selection signals between table and wine grapes during selective breeding. To this end, we randomly selected 30 wild accessions (*V. vinifera* ssp. *sylvestris*) and 90 domesticated grape accessions (*V. vinifera*) from a previous resequencing study (Liang et al., 2019). The domesticated grapes include 30 each in three sub-groups: wine, table and dual-purpose grapes. Using the available SNPs, we present phenotypic and genetic difference as well as selection signals during the domestication of wine and table grapes.

## Materials and Methods

### DNA Sequencing Data and Phenotypic Data of 120 Grapevines

A set of 90 *V. vinifera* accessions including 30 wine grapes, 30 table grapes and 30 dual-purpose grapes which can be used for both fresh consuming and wine production, as well as 30 wild *sylvestris* accessions were randomly selected from the samples of a previous work (Liang et al., 2019), in which we re-sequenced 472 *Vitis* accessions on an Illumina HiSeq 4000 sequencer at Novogene-Beijing. The raw sequencing data of these 120 accessions were obtained from NCBI database^1^ under BioProject PRJNA393611, totaling 728 Gb. Detailed information of these samples, including SRA ID, cultivar name, country of origin, ploidy level, breeding parents, etc. were listed in Supplementary Table 1. Utilization of all accessions were simultaneously checked in VIVC database^2^ and by experienced researchers.

Liang et al. (2019) reported in their study a total of 24 traits for the cultivated accessions at the ripening stage. We obtained the corresponding phenotypic data for these 90 selected accessions from this study. Three phenotypes (the amount of benzaldehyde, phenylethyl alcohol and 6-methyl-5-hepten-2-one) were discarded due to high proportion of missing value. In the end, a total of 21 traits were reserved in this study (Supplementary Table 2).

### Variation Calling and Annotation

Cleaned paired-end resequencing reads were mapped to the *Vitis vinifera* cv. PN40024 reference genome (Ensembl Plants Release-31, Jaillon et al., 2007) using BWA software (Version: 0.7.10-r789; Li and Durbin, 2009) with the default parameters. To convert mapping results into the BAM format and filter the unmapped and non-unique reads, SAMtools software (Version: 1.3.1; Li et al., 2009) was used. Duplicated reads were filtered with the Picard software (Version: 2.1.1; picard.sourceforge.net). After BWA alignment, the reads around INDELs were realigned by Genome Analysis Toolkit software (GATK, version 3.3-0-g37228af; McKenna et al., 2010) in two steps. In the first step, the RealignerTargetCreator function was used to identify regions where realignment was needed, then the IndelRealigner function was used to realign the regions to produce a realigned BAM file for each accession. Following the best practice workflow recommended by GATK, the variation detection was conducted. In brief, the variants were called for each accession using the GATK HaplotypeCaller function (Emanuelli et al., 2013). A joint genotyping step for comprehensive variations union was performed on the gVCF files. In the filtering step, the SNP filter parameter was set as “QD < 5.0 | | MQ < 540.0 | | FS > 60.0 | | SOR > 3.0 | | MQRankSum < −10.0 | | ReadPosRankSum < −8.0 | | QUAL < 30”. SNPs with MAF < 0.05 were further removed for phylogenetic tree structure, LD decay, PCA and population structure analysis. Making use of the ANNOVAR software (Version: 2015-12-14; Wang et al., 2010), SNPs annotation was performed according to the grapevine reference genome. The coverage of each accession against each chromosome was counted according to the aligned BAM files using SAMtools. SNP density and total genetic diversity across each chromosome were counted within a 100 kb sliding window by VCFtools software (v0.1.13; Danecek et al., 2011).

### Population Genetics Analysis

The whole-genome SNPs were used to construct the Maximum likelihood (ML) phylogenetic tree with 100 bootstrap using SNPhylo software (Version: 20140701; Clark et al., 2007). The iTOL^3^ (Letunic and Bork, 2019) tool was applied to color the phylogenetic tree. SNPs in linkage disequilibrium (LD) was filtered by PLINK software (Version v1.90b3.38; Purcell et al., 2007) with a window size of 50 SNPs (advancing 5 SNPs at a time) and an *r*^2^ threshold set to 0.5. Principal component analysis (PCA) was performed with the Genome-wide Complex Trait Analysis software (GCTA, version: 1.25.3; Yang et al., 2009, 2011), and the first three eigenvectors were plotted. To analyze the population structure, the ADMIXTURE program (Version: 1.3; Alexander et al., 2009) with a block-relaxation algorithm was applied. The convergence of individuals was explored by predefining the number of genetic clusters K from 2 to 4 and running the cross-validation error (CV) procedure. To calculate the linkage disequilibrium (LD), the PopLDdecay (Version: v3.31^4^) software was employed and the pairwise *r*^2^ values within and between different chromosomes were calculated. For each sub-group, the LD was calculated using the corresponding SNP pairs.

### Genome Scanning for Selective Sweep Signals

To detect selective sweeps, SweeD software (Version 3.3.1; Sheehan et al., 2013) was utilized based on the composite likelihood ratio (CLR) test to detect signatures of domestication in the table and wine accessions, respectively. To investigate the selection signals across the whole genome, we also calculated the population divergence statistic (F_ST_) and population nucleotide diversity (π, pairwise nucleotide variation as a measure of variability). A 100 kb sliding window with 10 kb step was applied to quantify F_ST_ and π by using VCFtools software (v0.1.13; Danecek et al., 2011). Sliding windows with both of the top 5% values were picked as candidate selective signals.

## Results

### Phenotypic Variation

In our study, a total of 120 grape accessions, including 90 domesticated grape accessions (30 each in wine, table and dual-purpose grapes) and 30 of its wild relative (*V. vinifera* ssp. *sylvestris*), were randomly selected. The geographic distributions of these accessions were from total of 23 countries, including China, France, Japan, the United States, Italy, etc. (Supplementary Table 1). When comparing the country of origin between each sub-group, a correlation between geography and utilization can be found. In our datasets, there were 28 table grape accessions with geographic data, of which 20 (71.4%) were Eastern table grapes but only 8 were Western (28.6%). In comparison, 84.6% (22) of the wine table accessions were Western and only 15.4% (4) were Eastern wine grapes. While for dual purpose accessions, less difference was observed between the proportion of Eastern (42.9%) and Western (57.1%). As an attractive trait for consumers, skin color showed different proportion in the three sub-groups. Among 30 table grape accessions, the majority were in white (11) and red (16) while only 1 and 2 were in pink and black, respectively. Dual-purpose grapes also had four colors, but more than half (17) were in white and 2, 3, and 8 were in pink, red and black, respectively. As for the wine grapes, there was no pink or red accession but 1 gray-skinned accession, and white and black skin-colored accessions almost had the equal number (12 and 13, respectively). Additionally, almost all of the cultivated accessions had hermaphrodite flowers except TA-334, which was a female accession.

Moreover, phenotypic data of 21 traits on 90 cultivated grape accessions (*V. vinifera*) were collected (Supplementary Table 2 and Supplementary Figure 1). All of these cultivated accessions were planted in the *Vitis* germplasm repository at the Institute of Botany of Chinese Academy of Sciences in Beijing and harvested at the ripening stage, which was determined by the browning seeds and stable sugar content. Berry weight can be an index referring to the berry size. Though there was a small berry accession (TA-261, whose average berry weight was 1.64 g), table grapes had an average berry weight (5.80 g) significantly larger than that of wine grapes (2.27 g). The berry weight of dual-purpose grapes was in between. Additionally, wine grapes tended to have rounder berry shape as the ratio of length/width was closer to 1. The table grape accession TA-268 had the biggest number of 1.698. Among 3 sub-groups, the average seed number seemed similar, but table and dual-purpose grapes did have seedless accessions. In addition, significant differences were also observed in the acid levels among table grapes (3.48 ± 0.72 mg/ml for tartaric acid, 1.49 ± 0.68 mg/ml for malic acid and 4.97 ± 1.10 mg/ml for total acid), dual-purpose grapes (4.32 ± 1.13 mg/ml for tartaric acid, 1.87 ± 0.97 mg/ml for malic acid and 6.19 ± 1.54 mg/ml for total acid) and wine grapes (4.74 ± 0.70 mg/ml for tartaric acid, 2.29 ± 0.89 mg/ml for malic acid and 7.03 ± 0.85 mg/ml for total acid), showing a higher acidity of wine grape. However, among three sub-groups the less significant difference in sugar level was unexpectedly observed. The similar range of sugar level was surprisingly detected between 0 and 8.42 mg/ml for sucrose and 47.36 and 211.83 mg/ml for total sugar. When comparing the brix concentration detected during 2015 and 2017, wine grapes were found to have higher variance. For instance, TA-291, its brix content was 23.41° in 2015 and 20.10° in 2017, but in 2016 this value was only 13.48°. TA-236 was another example, it had 10.85° brix in 2016 but 18.60° in 2017. Moreover, extreme low cases of brix were also found in wine grapes, such TA-242 (7.80° in 2015 and 7.50° in 2017) and TA-183 (9.99° in 2017). For aroma, uptrends could also be observed in the content of hexanal, 2-hexenal, nonanal and (E)-2-hexen-1-ol, indicating their essential role in high quality winemaking.

When considering the breeding history that referred to the parent-offspring relationship in our accessions, we found that at least one parent had the same usage as the offspring, i.e. breeding parents (at least one) should be the same grape type as the offspring or be dual-purpose. Additionally, several accessions were siblings sharing the same breeding parents, and particularly noteworthy was that even the same parents could breed offspring of different types (usage). For instance, Cabernet Suntory and Suntory Blanc are both bred from the cross between Koshu Sanjaku and Cabernet Sauvignon, but they were dual-purpose and wine grape, respectively. The same situation was found in Aishenmeigui (dual-purpose grape) and Zaomanao (table grape), whose parents are dual-purpose grapes Muscat Hamburg and Jingzaojing. Together, these observations could further evidence that grape is a highly heterozygous species with complex genetic background and quantitative agronomic traits.

### Genomic Variation

The sequencing data of 120 grape accessions (4.85 Gb reads) have an average coverage depth of 16.2X for each accession (Supplementary Table 3). After mapping against the *V. vinifera* reference genome (Jaillon et al., 2007), the average mapping rate was 98.17% with the genome coverage above 80% across all chromosomes for the majority of accessions (Supplementary Table 3). After a basic filtering criterion (minor allele frequency >0.05 and missing rate <40%, see section “Materials and Methods”), a total of 7,522,958 single-nucleotide polymorphisms (SNPs) were identified on 19 chromosomes (Supplementary Figure 2). Among 19 chromosomes, the largest number of SNPs (543,458) were identified on chromosome 14 with a moderate SNP density of 18 SNPs/kb while both of the lowest SNPs number (305,687) and also the lowest SNP density (∼16 SNPs/kb) were identified on chromosome 2. Additionally, chromosome 15 and 12 were two chromosomes with the densest SNP distribution with the density of approximately 19 SNPs/kb.

### Population Structure and Linkage Disequilibrium

**FIGURE 1:**
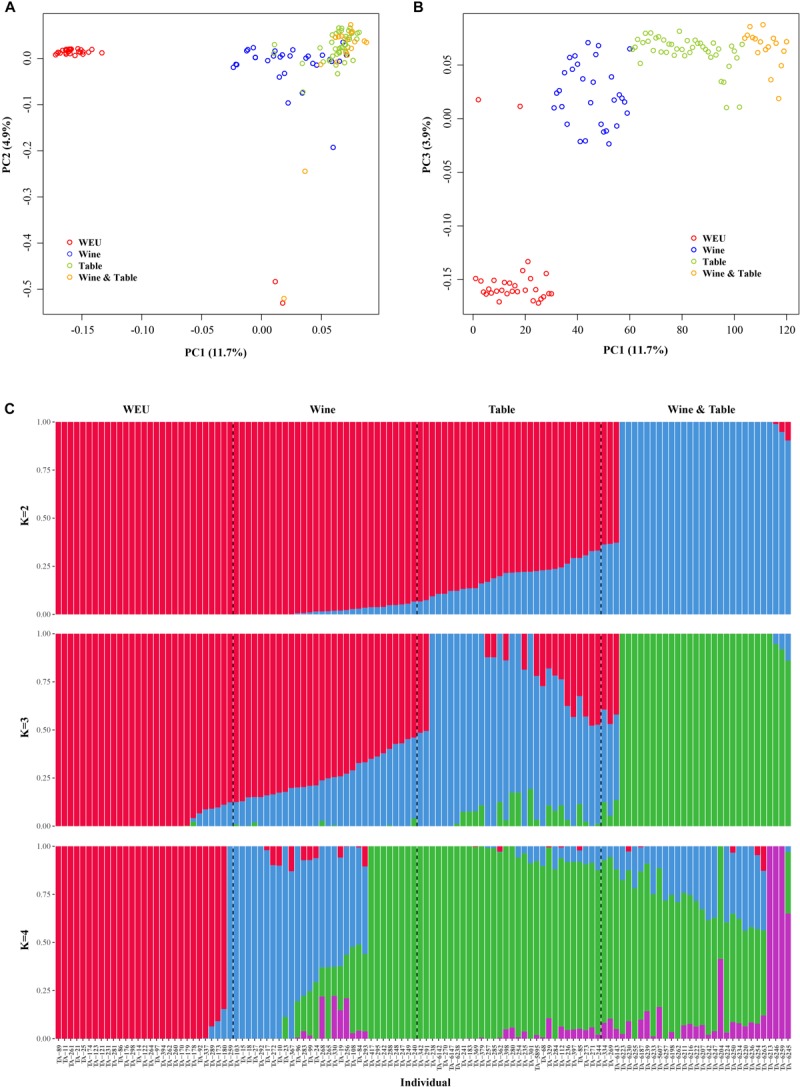
Population structure analyses of all accessions. **(A,B)** PCA analysis of all accessions in this study. **(C)** Population admixture of all Vitis accessions. Each color represents one ancestral population. Each accession is represented by a vertical bar, and the length of each colored segment in each vertical bar represents the proportion contributed by ancestral populations. WNA, Wine, and Table represent wild European grapevine, wine grape accessions, and table grape accessions, respectively.

**FIGURE 2:**
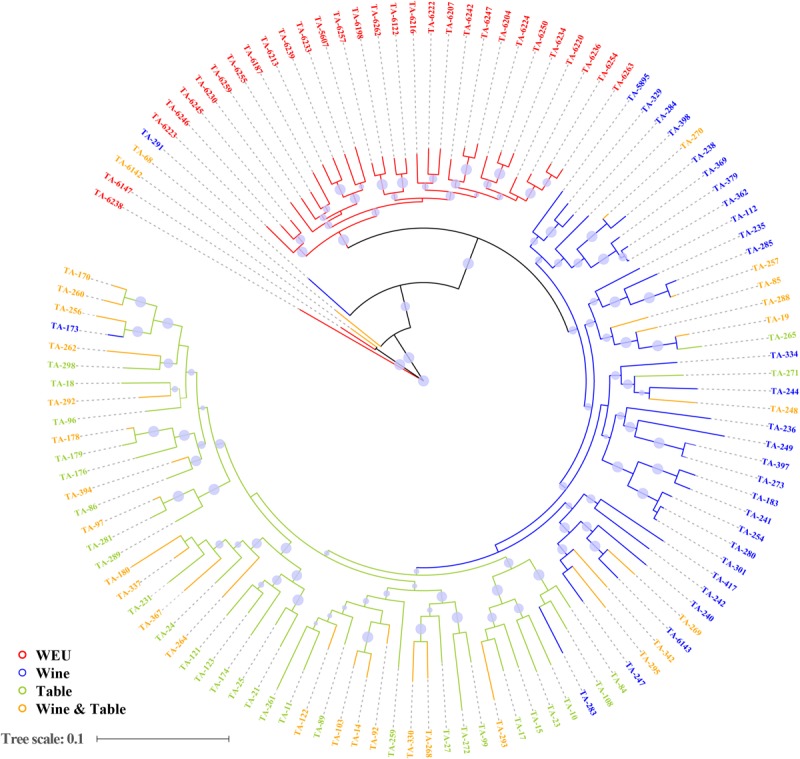
Maximum Likelihood phylogenetic tree of all accessions inferred from whole-genome SNPs. Red, blue, green, and orange represent wild European grapevines (WEU), wine grapes, table grapes and dual-purpose grapevines which can be used for both fresh consuming and wine production, respectively. Bootstrap values are indicated by blue circles.

In accordance with expectations, PCA showed substantial genetic diversity among major grapevine categories. 11.7, 4.9, and 3.9% of total genetic variance were explained by the first three principal components, respectively (Figures 1A,B). PC3 separated wild Eurasian accessions (WEU, *V. vinifera* subsp. *sylvestris*) from the cultivated grape accessions (table, wine and dual-purpose grapes, Figure 1B), supporting the fact that the latter three sub-groups shared more similarity in the genetic background than wild Eurasian accessions. This finding was further supported by the result of model-based analyses of population admixture (Figure 1C) and phylogeny analysis (Figure 2). PC1 evidently separated WEU, table grapes, wine grapes and dual-purpose grapes whereas PC2 set two WEU and one dual-purpose accessions apart from other accessions (Figure 1A). The differentiation between these grapevine categories was also consistent with the population admixture graph (Figure 1C, *K* = 4). In the admixture plot at *K* = 4 (Figure 1C), it was apparent that comparing to the table grapes, the majority of wine grapes received more genetic contributions from WEU, whereas table and dual-purpose grapes shared more similar genetic background and this result was in line with the ML phylogenetic tree (Figure 2). Even though most of domesticated grapevine accessions were closely clustered in the PCA graphs, they showed clear pattern of high genetic heterogeneity as evidenced by the population admixture analyses (Figure 1C, *K* = 4).

**FIGURE 3:**
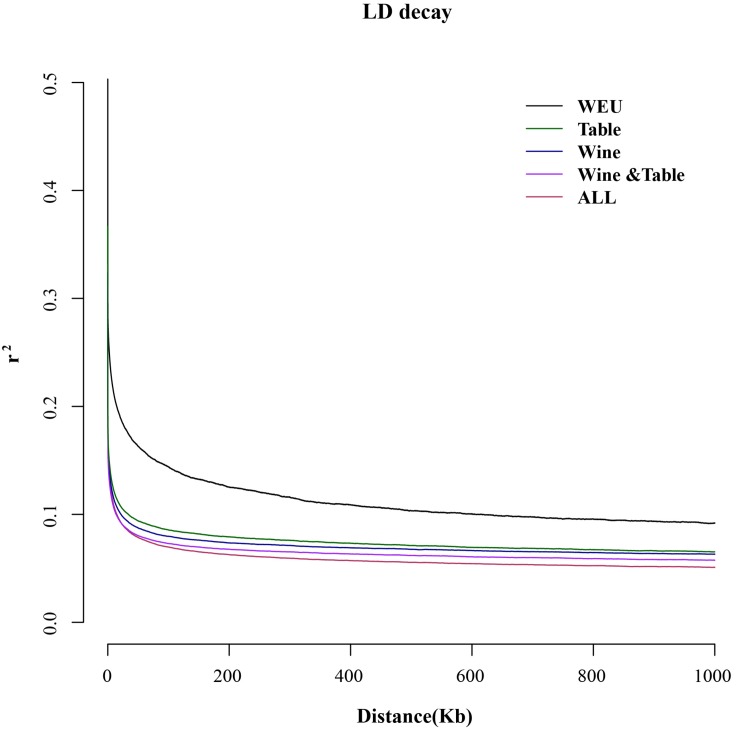
Decay of linkage disequilibrium in different sub-groups and different chromosome regions.

Linkage disequilibrium (LD) was evaluated in wild European and each domesticated sub-group using large-scale genetic markers (Figure 3). For wild European grapevines the LD decay reached half of the maximum average *r*^2^ at a distance of 3 kb while all three sub-groups of domesticated grapevines had similar LD around 350 bp, being concordant to the previous study (Marrano et al., 2018; Liang et al., 2019). Comparing with the domesticated grapevine accessions, the wild European species had relatively slower decay of LD, but it is important to realize that this difference may narrow with a more diverse wild European *Vitis* population. The LD correlates to the number of recombination events along generations, thus lower or higher LD within subgroups may allow to testify different group histories (Laucou et al., 2018). However, the calculation of LD is sensitive to the population size and the detected regions (Nicolas et al., 2016). In this study, the LD had not significant difference between three sub-groups of cultivated grapes. Thus, any conclusion of group history or possible causal relation of LD could not be taken.

### Selection Signals During Domestication of Wine and Table Grapes

**FIGURE 4:**
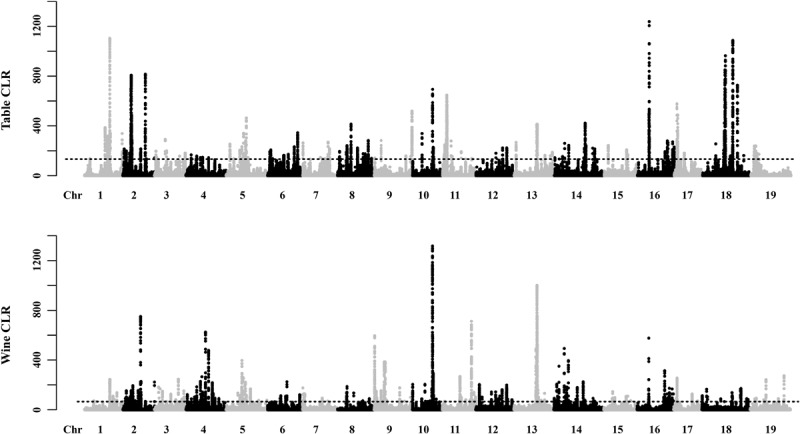
Global View of Candidate Domesticated Regions in the table and wine grapes. Regions with both XP-CLR values and p ratios in the top 5% were regarded as having domestication signals. The admixture patterns of all accessions are presented in Figure 1C. The sources of all the samples are provided in Supplementary Table 1.

**FIGURE 5:**
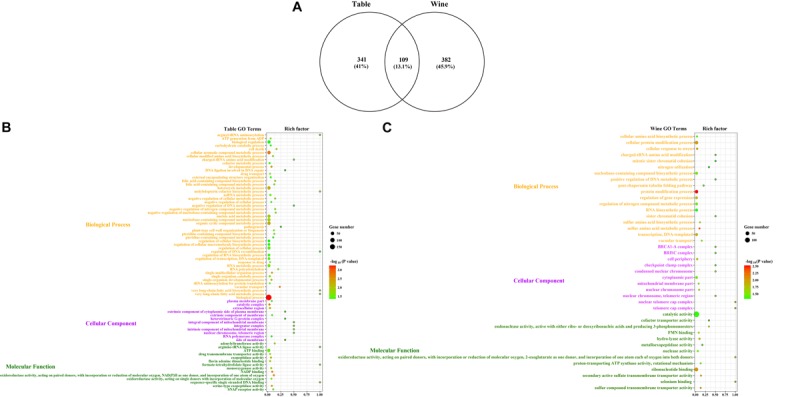
GO enrichment of the candidate selective sweep genes from the CLR analysis. **(A)** Venn plot of the GO terms shared by table and wine grapes. Bubble plots of enriched GO terms for **(B)** the table grapes (*n* = 30) and **(C)** wine grapes (*n* = 30). The size of the bubble represents the number of genes in the corresponding GO category. The color of the bubble shows the corresponding *P*-value. Rich factor shows the percentage of enriched genes out of the total number in the GO category.

The potential selective signals in the table and wine grapevines were investigated by identifying the regions (∼1 kb in length) that both scored in the top 5% of the Fst and CLR analysis (Figure 4, Supplementary Figures 3, 4 and Supplementary Tables 4, 5). 2,119 selective sweep regions were both detected in table and wine grapes, which harbored 450 and 491 candidate genes, respectively, and 109 overlapped genes (Supplementary Tables 4–6). For table grape accessions, the highest signal 1237.24 was found on chromosome 16 at position 6,961,106 bp, and the most selective signals were detected on chromosome 2 (358 signals) while chromosome 4 had the least signal number of 19. For wine grapes, 300 selective signals were identified on chromosome 13 but only 22 on chromosome 15, with the highest signal reached to 1317.4 on chromosome 10 at 12,113,058 bp. In addition to the 2 highest signals on chromosome 10 and 16, both table and wine grapevines shared some other high signal at the same position, including chromosome 1 (15,167,366 bp), chromosome 13 (13,996,633) and chromosome 2 (4,918,924 bp). It was worth mentioning that the signal on chromosome 2 at 4.92 Mb was within the fine-mapped 143 kb regions (4.91–5.05 Mb) believed to harbor the causal flower sex locus (Fechter et al., 2012; Picq et al., 2014). Meanwhile, some special signal for different grape accessions indicated they had undergone a different selection pressure, such as table grape unique signals on chromosome 18 at 18,447,039 bp and 21,205,860 bp and wine grape unique signal on chromosome 11 at 17,260,626 bp. Annotation of genes in selective sweep regions identified two (*VIT_12s0034g00310* and *VIT_18s0075g00100*) and three (*VIT_04s0043g00970*, *VIT_09s0002g06180*, *VIT_19s0015g01260*) disease resistance related genes specific for table and wine, respectively, indicating their undergoing of the different breeding process for disease resistance. Additionally, genes *VIT_16s0013g01070* and *VIT_16s0013g01080* on the flanking sides of the highest signal on chromosome 16 were annotated as ethylene-responsive transcription factor *ERF105* and *ERF104*, respectively. In *Arabidopsis*, *ERF104* and *ERF105* were found to play important role in signaling response, plant immunity and plant response to abiotic stresses (Mase et al., 2013; Meng et al., 2013; Vogel et al., 2014; Müller and Munné-Bosch, 2015; Cao et al., 2019). By inference, this positive selected region may relate to the stress tolerance of grapevine. Unfortunately, there were more candidate genes at or close those high signal positions had unclear molecular function, thus the proper trait selected during the domestication could not be deduced. GO enrichment of the 341 table grape unique and 382 wine grape unique domesticated candidate genes showed significant difference in functional representation in the GO categories (Figure 5), whereas table grapes had most of genes annotated to biological process and wine grapes had most related to catalytic activity, which plays a vital role during the synthesis of aroma components and winemaking.

## Discussion

In this research work, we collected 30 accessions each in table grapes, wine grapes, dual-purpose grapes and wild relative (*V. vinifera* ssp. *sylvestris*). Given that most table grape accessions were from the East and most wine accessions were from the West, we found a correlation between usage and geography, which is likely to be influenced by religions (Migicovsky et al., 2017). In East, the consumption of alcohol has been prohibited among Muslim countries for over a millennium, grape breeding has thus more focused on the development of table grapes with attractive traits like large berry size and colorful skin (This et al., 2006). Conversely, as a dominant religion in Western country, Christianity does not prohibit alcohol and grapes have been selected to produce the high-quality wine.

Differ from wine grapes which are pressed and fermented prior to consumption, table grapes are consumed directly. Therefore, their desirability depends largely on a visual assessment by consumers. As a result, most table grapes have large and colorful berries. Our results also confirmed that table grapes, even the dual-purpose grape accessions, had larger and colorful berries than wine grapes, indicating that these characteristics may have been targeted by table grape breeders. Smaller berries, by contrast, often have more better properties for vinification hence may be more desirable and suitable for winemaking (Gil et al., 2015). In this study, there had three table grape accessions and four dual-purpose accessions produce no seed, indicating that seedlessness becomes another preferable trait for fresh fruit consumption and probably for raisin production. Acidity also comes in to play when comparing these two grapes that higher acid levels were detected in wine grapes comparing with table grape, which was consistent with the previous work (Migicovsky et al., 2017). Sweetness is another characteristic where these two grape types should differ drastically. Wine grapes are commonly harvested at around 22–30 percent sugar whereas table grapes at around 15–20% sugar. This high sugar content is preferred because sugar is converted by yeast into alcohol by the process of fermentation. The greater concentration of sugars in grape berries there is, the greater potential alcohol level will be produced. For this reason, sugar content is a much more important factor for wine grapes. Some winemakers and viticulturists will continually sample grapes for a higher potential alcohol level and even delay harvesting time until grapes have a sufficiently high sugar concentration (Bird, 2011; Robinson and Harding, 2015). However, in our study, sugar content of grapevine, especially of wine grapes, showed a less reliable results. For instance, Cabernet Franc (i.e. TA-183), is usually harvested above 20° Brix (Smart et al., 1990; Gaudillère et al., 2002), but had only 9.99° Brix in 2017. This huge difference was possibly due to the unusual high temperatures in Beijing. In long terms, the effects of temperature on grapevine have been recognized. It influences plant physiology, berry composition and ultimately wine characteristics (Jones et al., 2005; Bonada and Sadras, 2015). Although the basic climatic conditions for grape growing are easily satisfied, high temperatures may make it difficult to consistently fulfill criteria required for grape quality without adjusting variety or changing management practices (Martínez-Lüscher et al., 2016). Consequently, the content of sugars and probably other phenolic compounds show very different accumulation patterns throughout grape development.

The genetic structure of grapevine, which can be largely understood as one large complex pedigree, is the result of a limited number of crosses among elite cultivars (Myles et al., 2011; Bacilieri et al., 2013). In this study, we randomly selected 90 cultivated grape accessions (30 each for different usages) for the identification of selective sweep, but common breeding parents were found between several accessions, some of which were even in the different sub-groups. This relatedness of genetic background may reduce the identification scope, but on the other hand had more precise detection of the selected signals. Due to the limited number (30) of each genetic pool, we are unable to provide any conclusion of group history with the current dataset, but still, the rapid LD decay in cultivated grapevine, is far more quickly than that in rice (Mather et al., 2007; Huang et al., 2012), soybean (Zhou et al., 2015), and *Arabidopsis* (Kim et al., 2007).

Positive selection acts on beneficial alleles, increase their frequency in the population and leaves signature over time in the genome. The analysis of large genomic data allows the detection of the molecular patterns of advantageous mutations that have been selected and fixed during domestication and breeding. In this study, an SFS-based method (SweeD) with the composite likelihood ratio (CLR) test was applied in table and wine grape accessions, and each had 2119 positively selected signals, covering 450 candidate genes and 491 candidate genes, respectively. Among them, a signal on chromosome 2 at the position of 4.91 Mb was shared by both groups, where the sex-determining locus had been fine-mapped (Fechter et al., 2012; Picq et al., 2014). As one of the oldest cultivated fruits, the most discriminating characteristic between the cultivated *V. vinifera* ssp. *vinifera* and the wild-form *V. vinifera* ssp. *sylvestris* is their sexual system. Flowers of cultivated grapes are mainly hermaphroditic, whereas all wild *Vitis* species, including the ancestor of *V. vinifera*, are dioecious. This key transition enables self-pollination and subsequent clonal propagation without the need for pollinators (Palumbo et al., 2019).

Due to the advancement of high-throughput sequencing technology (Alaimo et al., 2017), a huge number of QTLs underlying important phenotypic traits have been explored, such as the MADs-Box gene *VviAGL11* on chromosome 18 which codes seedlessness (Mejia et al., 2011; Royo et al., 2018), *VvMybA1* on chromosome 2 that affects grape skin color, and several other traits like berry firmness (Carreño et al., 2014; Correa et al., 2016) and resistance (van Heerden et al., 2014; Pap et al., 2016; Zyprian et al., 2016; Zendler et al., 2017; Hou et al., 2018). However, the highest 4 positive selection signals shared by both table and wine grape, which were chromosome 1: 15,167,366 bp, chromosome 10: 12,113,058 bp, chromosome 13: 13,996,633 bp and chromosome 16, 6,961,106 bp, were not linked to those QTLs. Nevertheless, two flanking genes at 6,961,106 bp on chromosome 16 were annotated as *ERF104* and *ERF105*, respectively, which were involved in the immunity and stress response in *Arabidopsis*. Thus, we may deduce that the candidate genes at or close at these positions could be very important genes related to the traits selected during the domestication process, such as stress response.

During domestication, the selection is often aimed at some specific phenotype, where the internal mechanism is to select genes that have a direct relationship with the preferable traits. In this study, we have identified several genomic regions under positive selection that may have been artificially selected during the process of grapevine domestication. The detection of these significant selection regions can lead to the candidate genes that perform the corresponding functions and therefore would have great significance to understand the evolution of grapevine (Adam-Blondon et al., 2005; Lamoureux et al., 2006). Economically speaking, the improvement of wine quality and environmental stress resistance during domestication by genetically artificial screening has been making great benefits to both of the wine industry and fresh fruit market.

## Data Availability Statement

The WGRS data set generated and analyzed in the current study is available from NCBI under the BioProject accession PRJNA393611.

## Author Contributions

LK collected the samples and performed the experiments. SD and SW completed the data analysis. XD and MT edited and modified the manuscript. All authors read and approved the manuscript.

## Conflict of Interest

SW, SD, and XD are employees of the Nowbio Biotechnology Company and they declare that Nowbio Biotechnology Company plays no role in the funding, design, analysis, and publication of this manuscript.

The remaining authors declare that the research was conducted in the absence of any commercial or financial relationships that could be construed as a potential conflict of interest.

## Footnotes

1 http://www.ncbi.nlm.nih.gov/Traces/sra

2 http://www.vivc.de/

3 http://itol.embl.de

4 https://github.com/BGI-shenzhen/PopLDdecay

## Abbreviations

PCA: principle component analysis
QTL: quantitative trait locus.
