# Supplementary figures and images for: Identification of Selective Sweeps in the Domesticated Table and Wine Grape (Vitis vinifera L.)
Source: Front Plant Sci. 2020 May 14;11:572. doi: 10.3389/fpls.2020.00572 (PMC7240110; doi:10.3389/fpls.2020.00572)

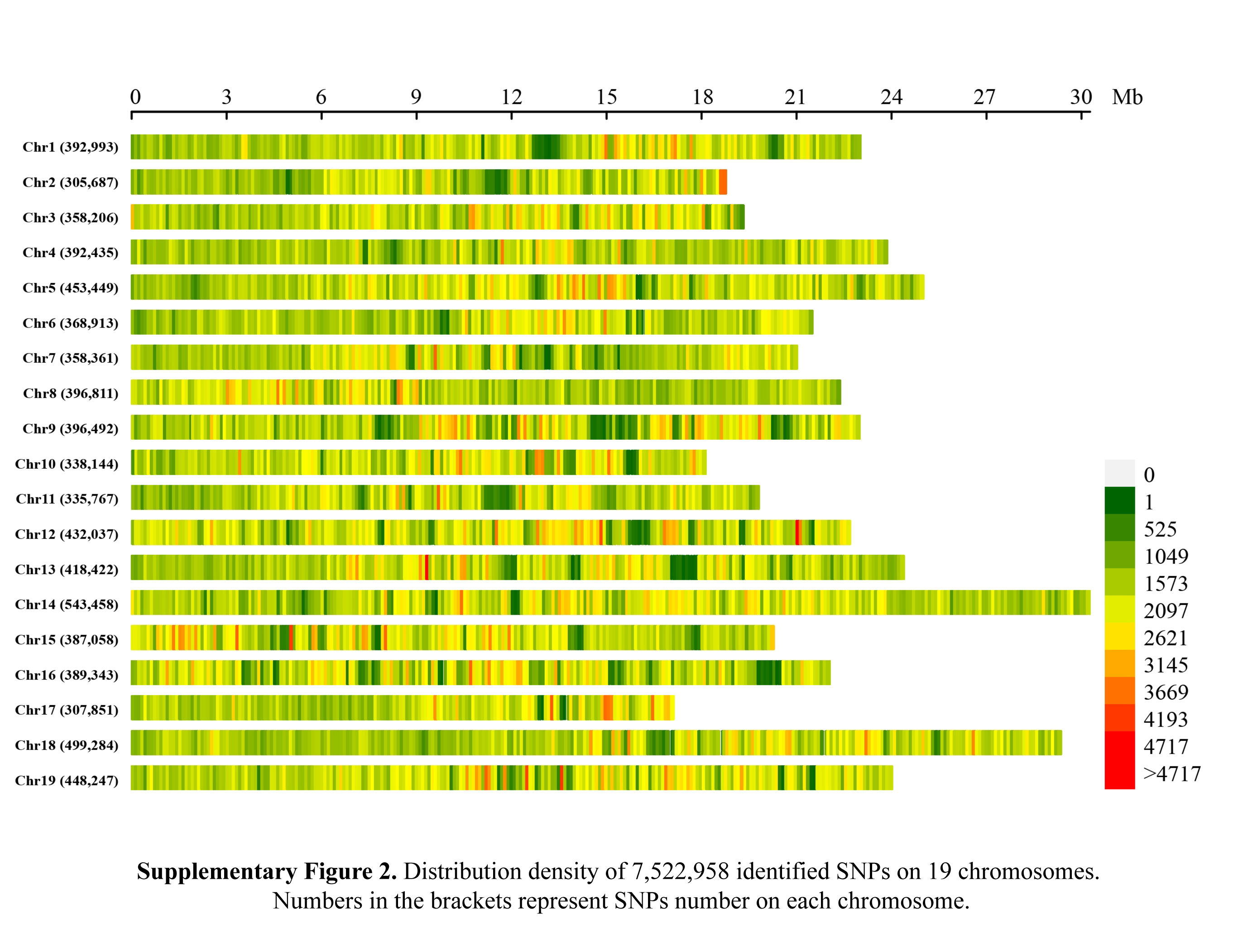

Supplement: Supplementary file 3 [file Image_2.jpg]

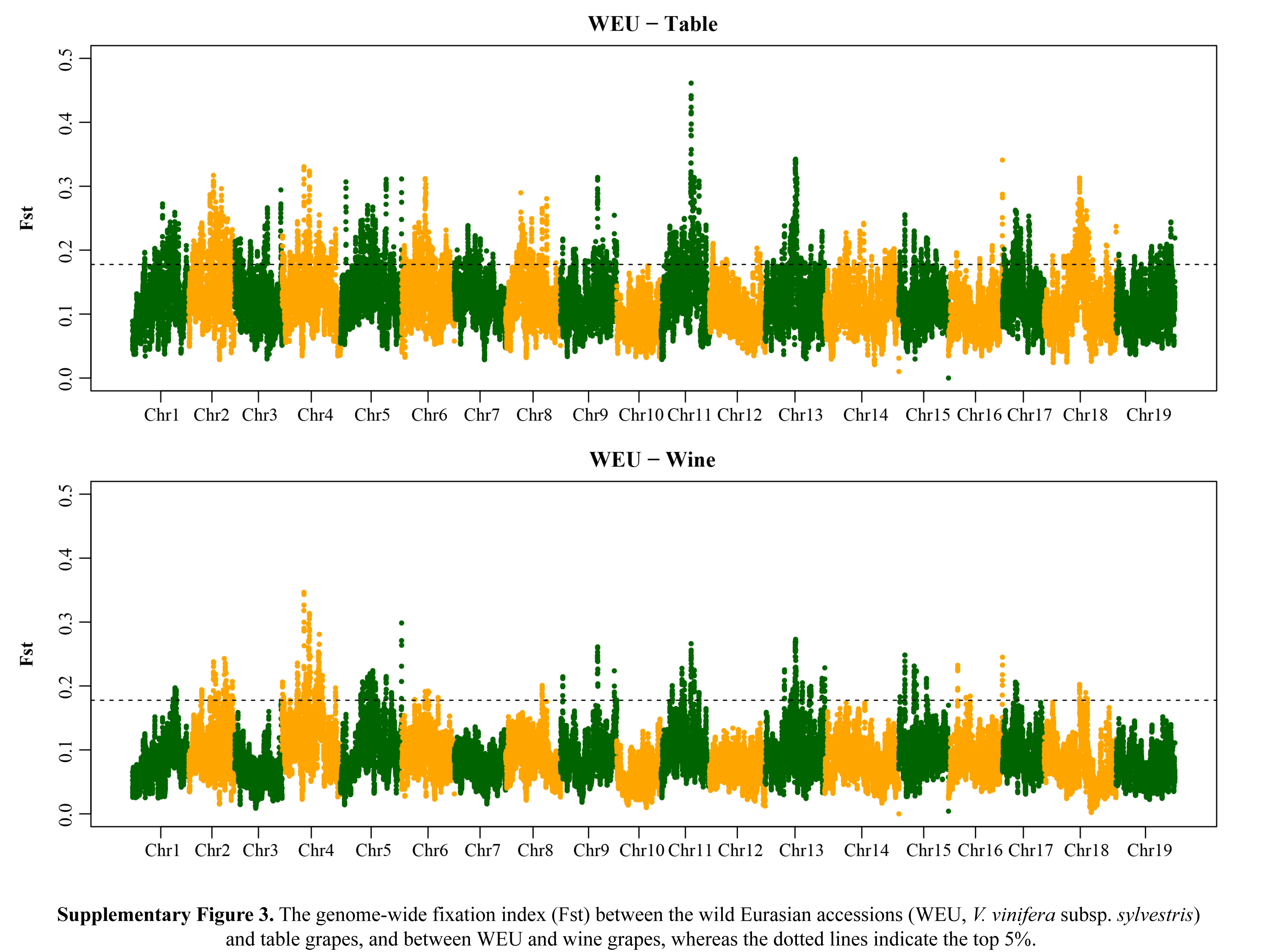

Supplement: Supplementary file 4 [file Image_3.jpg]

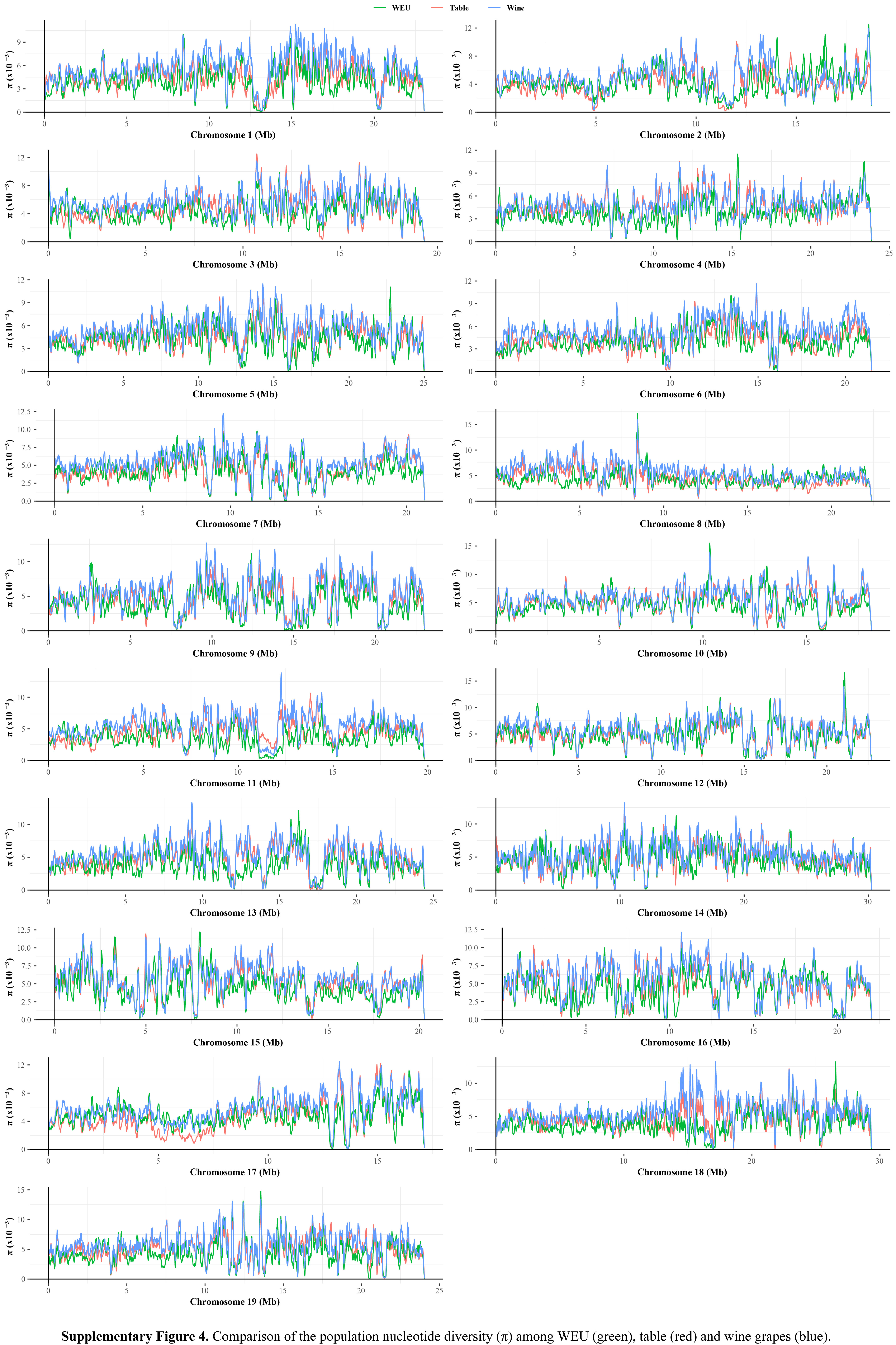

Supplement: Supplementary file 5 [file Image_4.jpg]
